# A comparative evaluation of intranasal α2-adrenoceptor agonists and intranasal midazolam as premedication in pediatric sedation: A meta-analysis of randomized controlled trials

**DOI:** 10.1371/journal.pone.0281751

**Published:** 2023-02-14

**Authors:** Yuzhi Fu, Qianqian Zhang, Yongxian Jiang, Bingchen Lang

**Affiliations:** 1 Department of Pharmacy, West China Second University Hospital, Sichuan University, Chengdu, China; 2 Evidence-Based Pharmacy Center, West China Second University Hospital, Sichuan University, Chengdu, China; 3 Key Laboratory of Birth Defects and Related Diseases of Women and Children, Ministry of Education, Sichuan University, Chengdu, China; 4 Department of Anesthesiology, Chengdu Women and Children’s Central Hospital, Chengdu, China; 5 Department of Pharmacy, Sichuan Provincial Maternity and Child Health Care Hospital, Chengdu, China; Sorbonne University Pierre and Marie Curie Campus: Sorbonne Universite Campus Pierre et Marie Curie, FRANCE

## Abstract

**Background:**

Midazolam and α2-adrenoceptor agonists have been widely used off-label as intranasal sedatives for children. The present meta-analysis aimed to evaluate the effects of two interventions in pediatric sedation.

**Methods:**

PubMed, Embase, and Cochrane Library were searched from inception to April 2022. All randomized controlled trials used intranasal α2-adrenoceptor agonists and midazolam as sedatives in children were enrolled. Parental separation, anesthesia induction or facemask acceptance, sedation level, different hemodynamic parameters and adverse events were considered as outcomes.

**Results:**

Totally 21 studies with 1,495 patients were included. Only one study reported comparison between midazolam and clonidine met the inclusion criteria, and patients in clonidine group had significantly better mask acceptance compared to midazolam group. Compared with midazolam, using of dexmedetomidine was associated with higher rate of satisfactory parental separation (52.88% vs 75.18%, RR = 0.70, with 95%CI [0.55, 0.90]), anesthesia induction or facemask acceptance (60.92% vs 81.47%, RR = 0.76, 95% CI [0.68, 0.84]) and less incidence of postoperative pain and nasal irritation.

**Conclusion:**

Compared with midazolam, dexmedetomidine should be considered as the preferred intranasal sedative option for pediatric patients, since it provides more satisfactory sedative level with less incidence of several side effects. But insufficient evidences about effects of intranasal clonidine and overall low and moderate quality evidences evaluated by GRADE system indicate that superiority of intranasal α2-adrenoceptor agonists in pediatric sedation needs to be validated by more studies with high quality and large sample size in future.

## Introduction

Alleviating anxiety and stress in pediatric patients before general anesthesia, diagnosis and procedures such as dental treatment and suturing is served as one major concern for anesthesiologist and pediatric clinicians. It has been reported that about 70% of children suffered from anxiety or fear during perioperative period and diagnostic procedures [1]. With appropriate sedatives, uncooperative physically resistance and emotional discomforts from children can be reduced before parental separation, diagnostic procedure, and induction of anesthesia [2].

As a short-acting benzodiazepine, midazolam provides fast sedation and promising recovery profile with relatively slight fluctuation in hemodynamic status, and its active metabolite “1-hydroxymidazolam” contributes to depression of neuronal activity [3]. According to a growing number of studies, α2-adrenoceptor agonists (such as dexmedetomidine and clonidine) have been shown to be effective in facilitating anesthetic induction and alleviating presurgical or pre-procedural anxiety for children [4–6]. Compared with clonidine, dexmedetomidine has an eight-fold greater affinity for α2 adrenergic receptors. Owing to higher selectivity for α2A receptors and shorter half-life, dexmedetomidine was considered to be more responsible for the sedative and analgesic effects than clonidine [7].

However, physiological feature (small veins and excess subcutaneous fat) of children often leads to difficulties in obtaining access to blood vessels during venipuncture. It requires several attempts and may cause stress for children in actual sedative process frequently [8]. Therefore, to increase compliance of children undergoing sedative premedication, nasal drug administration, an alternative route for intravenous administration without requiring venous puncture [9], has been applied in the field of pediatric sedation during the latest decades.

Recent comparative studies indicated that midazolam and α2-adrenoceptor agonists have been widely used as intranasal sedatives for children, and both of them merit particular attention. However, to our knowledge, no relevant systematic review has been established to compare the effects between two main approaches via intranasal route in pediatric sedation. Therefore, the aim of present study is to evaluate the efficacy and safety of two mentioned-above pharmacological approaches in children.

## Methods

### Search strategy

The present meta-analysis was performed in accordance with the recommendations in the Preferred Reporting Items for Systematic Reviews and Meta-Analyses (PRISMA) statement [10] and the guidelines described in the Cochrane Handbook. The protocol was registered with PROSPERO (CRD42022326979).

Two independent authors (YF and BL) searched PubMed, Embase, and Cochrane Library databases up to April 20, 2022. Moreover, we considered potentially useful studies in Google Scholar as additional source of information. The search terms we used included infant, child, adolescent, midazolam, nasal, intranasal and randomized controlled trial (S1 File). Only human studies published in English and Chinese were involved.

### Eligibility criteria

The studies meeting the following criteria were selected for further analysis:

#### Participants

Children (<18 years old) who experienced various presurgical and pre-procedural sedation.

#### Intervention and comparison

Using midazolam vs α2-adrenoceptor agonists via intranasal route as the sedative premedication.

#### Outcome measures

Satisfactory parental separation, anesthesia induction or facemask acceptance, stable hemodynamic status and limited adverse effects were considered as the features of ideal pediatric sedation [11], therefore, the co-primary outcomes in present study including: Number of patients with satisfactory separation from parents, number of patients with satisfactory induction or mask acceptance, and patient’s level of sedation. Moreover, onset of sedation, recovery time, general hemodynamic parameters, incidence of postoperative pain needed analgesics rescue and various adverse effects between two groups were also considered as the secondary outcomes.

#### Study design

In consideration of the quality of evidence, only randomized controlled trials (RCTs) were considered.

#### Exclusion criteria

Reviews, conference abstracts, cases, comments, preclinical studies, protocol, ongoing trials, studies not published in English or in Chinese, and studies with inappropriate comparisons or unrelated outcome measures were not considered.

### Data extraction, and assessment of the risk of bias

Two authors (BL and YF) conducted literature screening and data extraction individually, and they crosschecked with each other. After removing the duplicated studies from different databases, the irrelevant records were excluded by titles and abstracts screening. The full texts of the remaining studies were obtained and perused. And we designed a table to collect relevant details of all enrolled studies (Table 1). According to Cochrane risk of bias tool [12], two authors (BL and YF) independently evaluated the methodological quality including the following domains: random sequence generation (generation of the randomization sequence), allocation concealment, blinding of outcome assessment, incomplete outcome data, and selective reporting. All articles could have the following domain classifications: high risk of bias, low risk of bias, uncertain risk (without information for judgment). Any disagreements were resolved by consulting a third investigator.

**Table 1:** The general characteristics of the enrolled studies.

Study (Reference)	Year	Type of surgery /procedure	Patient age range & ASA status	Patients enrolled (Gender: F/M, n)	Intranasal Midazolam dose	Intranasal α2-adrenoceptor agonists dose	Scale used for sedation measurement	Outcomes
**Midazolam *versus* Clonidine**								
Mitra S *et al*. [14]	2014	Minor elective surgical procedures	1–10 yr, ASA I-II	60 (10/50) 1. Midazolam patients: 30 2. Clonidine patients: 30	0.3mg/kg	4μg/kg	6-points scale	I(b,c), VI (e)
**Midazolam *versus* Dexmedetomidine**								
Sundaram ALM *et al*. [15]	2011	Elective full mouth rehabilitation	2–9 yr, ASA I	80 (40/40) 1. Midazolam group: 40 2. Dexmedetomidine group: 40	0.2mg/kg	1μg/kg	6-points scale	I(a,b), III, V
Akin A *et al*. [16]	2012	Elective adenotonsillectomy	2–9 yr, ASA I	90 (37/53) 1. Midazolam patients: 45 2. Dexmedetomidine patients: 45	0.2mg/kg	1μg/kg	6-points scale	I(a,b), II, V, VI(a,b,c)
Mostafa G *et al*. [17]	2013	Bone marrow biopsy and aspirate	2–8 yr, ASA II	64 (Not mentioned) 1. Midazolam group: 32 2. Dexmedetomidine group: 32	0.2mg/kg	1μg/kg	4-points scale	I(a), V
Surendar MN *et al*. [18]	2014	Dental treatment	4–14 yr, ASA I	42 (Not mentioned) 1. Midazolam patients: 21 2. Dexmedetomidine patients: 21	0.2mg/kg	1μg/kg	5-points scale	I(c), III, IV
Sheta SA *et al*. [19]	2014	Complete dental rehabilitation	3–6 yr, ASA I-II	72 (41/31) 1. Midazolam patients: 36 2. Dexmedetomidine patients:36	0.2mg/kg	1μg/kg	4-points scale	I(a,b), II, III, IV, V, VI(a,b,d,e)
Singla D *et al*. [20]	2015	Elective surgery	3–10 yr, ASA I	60 (29/31) 1. Midazolam patients: 30 2. Dexmedetomidine patients:30	0.5mg/kg	1μg/kg	6-point scale	I(a,b), V
Patel DD *et al*. [21]	2015	Minor general surgical procedures	2–6 yr, ASA I-II	70 (Not mentioned) 1. Midazolam group: 35 2. Dexmedetomidine group: 35	0.3mg/kg	1μg/kg	6-point scale	I(a), II
Abdelaziz HMM *et al*. [22]	2016	Elective strabismus surgery	1–7 yr, ASA I-II	66 (32/34) 1. Midazolam patients: 33 2. Dexmedetomidine patients:33	0.1 mg/kg	1μg/kg	Not mentioned	II, VI(a,b)
Neville DN *et al*. [23]	2016	Suturing for lacerations	1–5 yr, ASA I-II	38 (13/25) 1. Midazolam patients: 18 2. Dexmedetomidine patients:20	0.4 mg/kg	2μg/kg	5-points scale	I(c)
Fei *et al*. [24]	2017	Surgery for pediatric tumors	1–3 yr, ASA I-II	60 (24/36) 1. Midazolam patients: 30 2. Dexmedetomidine patients:30	0.2mg/kg	1μg/kg	4-points scale	I(a,b), IV, V
Gupta A *et al*. [25]	2017	Elective brain magnetic resonance imaging	1–8 yr, ASA I-II	60 (26/34) 1. Midazolam patients: 30 2. Dexmedetomidine patients: 30	0.2mg/kg	1μg/kg	6-points scale	I(a,b), V
Messeha MM *et al*. [26]	2018	Cardiac catheterization	3–6 yr, ASA I-II	60 (31/29) 1. Midazolam patients: 30 2. Dexmedetomidine patients: 30	0.2mg/kg	0.1μg/kg	6-points scale	I(c), V
Azizkhani R *et al*. [27]	2020	Computerized Tomography	1–6 yr, ASA I-II	143 (46/97): 1. Midazolam patients: 65 2. Dexmedetomidine patients: 78	0.3mg/kg	3μg/kg	6-points scale	III, IV, V
Diwan G *et al*. [28]	2020	Elective surgery	2–12 yr, ASA I-II	60 (27/33): 1. Midazolam patients: 30 2. Dexmedetomidine patients: 30	0.2mg/kg	1μg/kg	6-points scale	I(a), V
Ghosh A *et al*. [29]	2020	Elective surgery	2–8 yr, ASA I-II	90 (20/70) 1. Midazolam patients: 48 2. Dexmedetomidine patients:42	0.5mg/kg	2μg/kg	5-points scale	I(b)
Saad BB *et al*. [30]	2020	Adenotonsillectomy	3–7 yr, ASA I	48 (19/29) 1. Midazolam group: 24 2. Dexmedetomidine group: 24	0.2mg/kg	1μg/kg	6-points scale	I(b,c), V, VI(b)
Panda S *et al*. [31]	2021	Transthoracic Echocardiography	1 mo-3 yr, ASA I-II	100 (35/65): 1. Midazolam patients: 50 2. Dexmedetomidine patients: 50	0.2mg/kg	2μg/kg	6-points scale	III, IV, V
Gupta A *et al*. [32]	2021	Elective surgery	2–8 yr, ASA I-II	70 (34/36) 1. Midazolam patients: 35 2. Dexmedetomidine patients: 35	0.2mg/kg	1μg/kg	5-points scale	I(b), V
Abusinna RG *et al*. [33]	2022	Minor elective surgical procedures	2–9 yr, ASA I-II	100 (54/46) 1. Midazolam patients: 50 2. Dexmedetomidine patients: 50	0.2mg/kg	1μg/kg	6-points scale	I(c), III, V
Agarwal S *et al*. [34]	2022	Routine surgical	1–5 yr, ASA I-II	62 (9/53): 1. Midazolam patients: 31 2. Dexmedetomidine patients: 31	0.5mg/kg	1μg/kg	Not mentioned	V

Note: I—Number of patients with (a. satisfactory separation from parents, b. satisfactory induction or mask acceptance, c. satisfactory sedation level); II—Incidence of postoperative pain needed analgesics rescue; III—Onset of sedation; IV—Recovery time; V—Hemodynamic status; VI—Adverse effects (a. Nauseas and vomiting; b. Agitation; c. Laryngospasm; d. Shivering; e. Nasal irritation/discomfort.)

### Grading the quality of evidence

The Grading of Recommendations Assessment, Development, and Evaluation (GRADE) methodology [13] was used to assess the quality of evidence and strength of recommendations. On the basis of risk of bias, inconsistency, indirectness, imprecision, and publication bias, the quality was classified as high, moderate, low, or very low. And GRADE profiler (version 3.6) software was used for evaluation.

### Statistical analysis

All statistical analyses were conducted using Review Manager software (Version 5.3.3, the Cochrane Collaboration 2014, the Nordic Cochrane Centre). Mean difference (MD) with 95% confidence interval (CI) was chosen to estimate continuous variables, and risk ratio (RR) with 95% confidence interval (CI) and the Mantel–Haenszel method (fixed or random models) were chosen to analyze dichotomous data. The *I*-squared (*I*^2^) test was used to weigh impact of heterogeneity on the results. If significant heterogeneity (present at *I*^2^ > 50%) existed, the sensitivity analysis was performed by omitting each study individually, and the random effects model was considered; otherwise, the fixed-effects model was used. And we used Begg’s test and Egger’s test to evaluate the publication bias if the number of included studies exceeds 10. Evaluation was conducted using version 1.2.4 of the metabias program, Stata/MP 12.0 for Windows (StataCorp LP, 4905 Lakeway Drive, College Station, TX 77,845, USA). A *P* value < 0.05 was considered statistically significant.

## Results

### Literature search results

**Fig 1:**
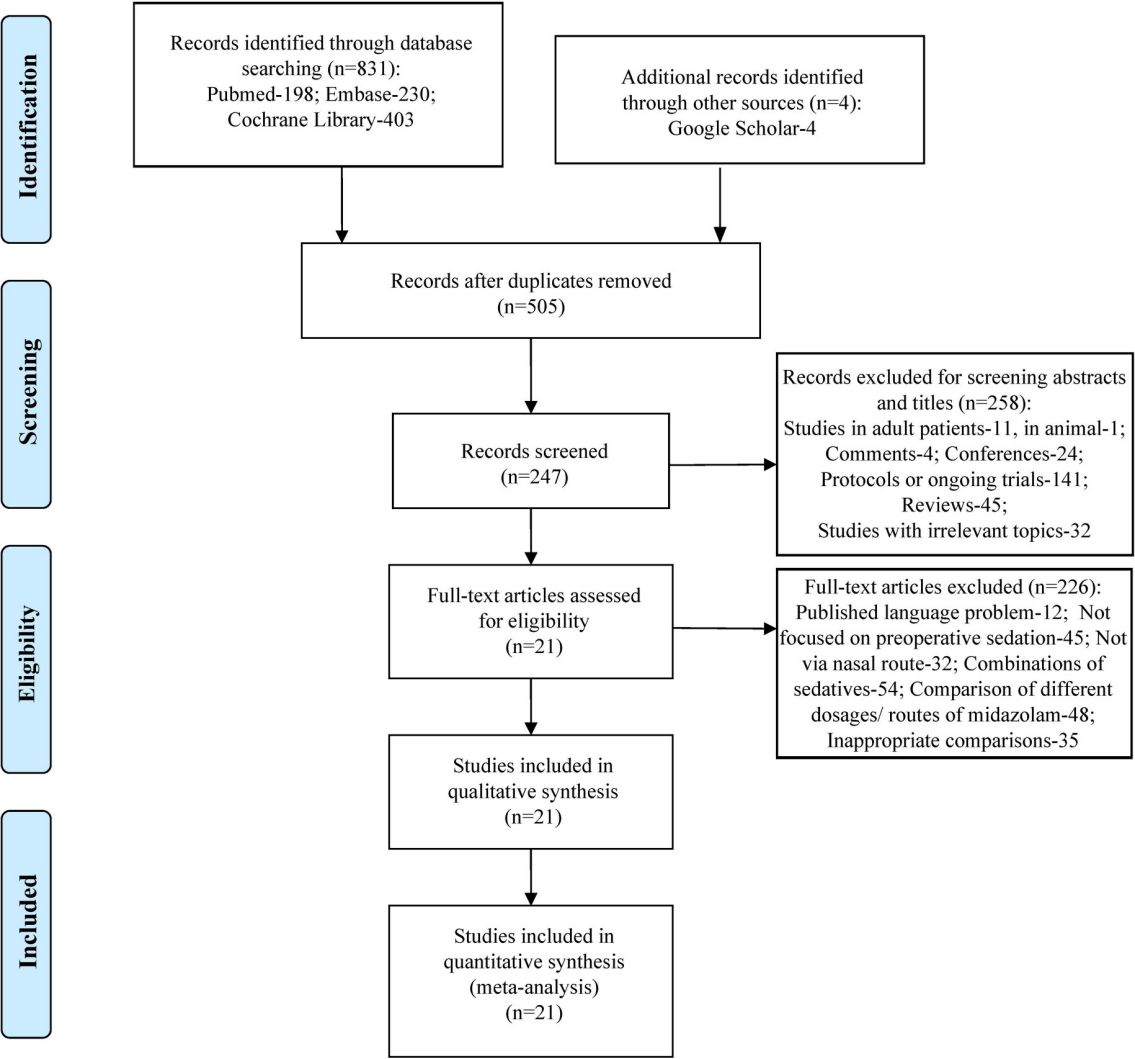
Flow chart of literature screening and the selection process.

After screening the databases and additional source, a total of 835 relevant items were identified initially. 330 duplicate records were removed, and 258 records were excluded by titles and abstracts reviewing. In these 258 excluded items, 11 were studies focused on adult patients, 1 was animal study, 4 were comments notes, 24 were conference abstracts, 141 were protocols or ongoing registered trials, 45 were reviews, and 32 were studies with irrelevant topics. And then 226 items were excluded by full-text screening, 12 of them were published neither in English nor in Chinese, 45 of them not focused on preoperative sedation, 32 of them reported the sedatives not via nasal route, 54 of them used combinations of sedatives, 48 of them focused on comparison of different dosages and different routes of midazolam, 35 of them were owing to the inappropriate comparisons. Finally, 21 studies were chosen in consequent analysis [14–34]. The process of literatures identification is described in PRISMA flowchart (Fig 1).

### Basic characteristics of enrolled studies

The included studies were published from 2011 to 2022, and a total of 1,495 eligible pediatric patients (ages ranged from 1 month to 14 years) were involved in present analysis. The primary outcomes “number of patients with satisfactory separation from parents, number of patients with satisfactory induction or mask acceptance, and patient’s level of sedation” were reported in 17 studies [14–21, 23–26, 28–30, 32, 33]. And most of studies paid attention on evaluation in midazolam versus dexmedetomidine, only one study focused on comparison between midazolam and clonidine [14]. The main characteristics of all enrolled studies were summarized in Table 1.

### Risk of bias assessment

**Fig 2:**
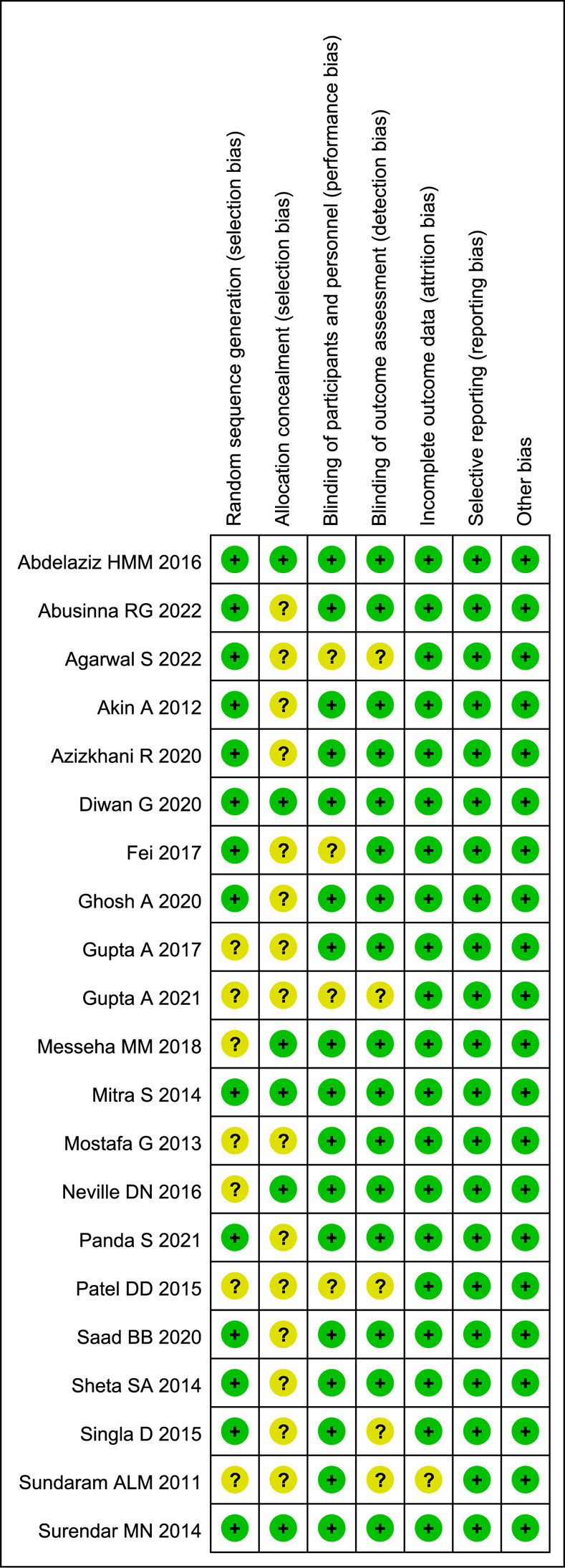
Risk of bias assessment of included studies. Green + dot, low risk of bias; yellow? dot, unclear risk of bias; red—dot, high risk of bias.

The validity and quality of enrolled RCTs were evaluated by using Cochrane Collaboration’s risk of bias tool [11]. A total of 66.67% (14/21) studies had low risk in random sequence generation domain, only 28.57% (6/21) studies mentioned the allocation concealment, 80.95% (17/21) studies described blinding procedure of participants and personnel, and 76.19% (16/21) studies described blinding procedure of outcome assessment. The details of risk of bias assessment were shown in Fig 2.

### Primary outcomes

#### Number of patients with satisfactory separation from parents

**Fig 3:**
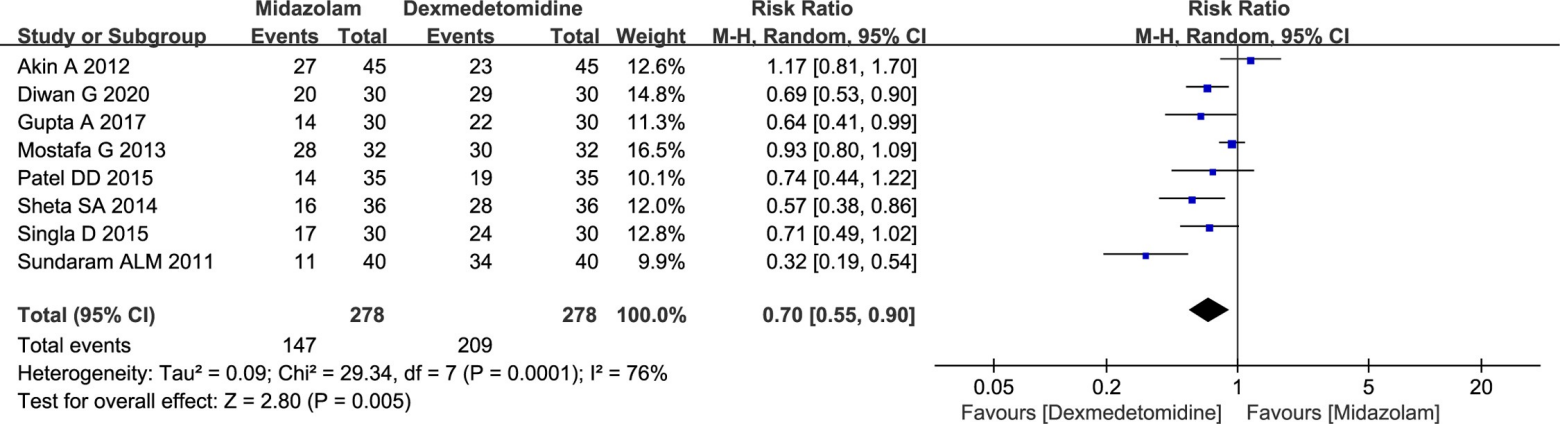
Forest plot depicting the meta-analysis for the outcome “number of patients with satisfactory separation from parents” for intranasal midazolam versus intranasal dexmedetomidine.

Eight studies including 556 pediatric patients reported the number of patients with satisfactory separation from parents, and all of them focused attention on comparison between midazolam and dexmedetomidine. The *I*^2^ of 76% denoted existence of substantial heterogeneity, however, the source could not be attributed clearly to one particular study by sensitivity analysis; therefore, the random effects model was applied. According to the result, intranasal dexmedetomidine was associated with more satisfactory separation from parents compared to intranasal midazolam (52.88% vs 75.18%, RR = 0.70, with 95%CI [0.55, 0.90], *P* = 0.005, *I*^2^ = 76%; Fig 3). According to GRADE summary of findings table, the quality of evidence for this outcome was moderate. It might be resulted from inconsistency (*I*^2^ > 50%) (S1 Table).

#### Number of patients with satisfactory induction or mask acceptance

**Fig 4:**
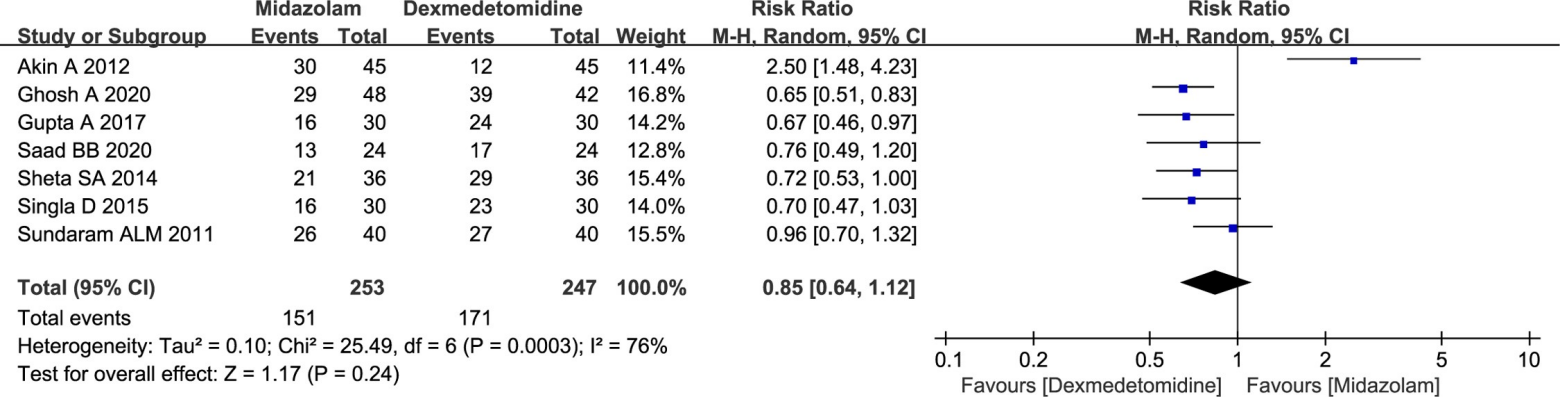
Forest plot depicting the meta-analysis for the outcome “number of patients with satisfactory induction or mask acceptance” for intranasal midazolam versus intranasal dexmedetomidine.

The results of Mitra S *et al*. [14] study indicated that patients in clonidine group had significantly better mask acceptance compared to midazolam group (100% *vs* 80%, *P* = 0.024). And a total of 500 children in seven studies paid attention on comparison between midazolam and dexmedetomidine. The using of dexmedetomidine was associated with slightly higher rate of satisfactory induction or mask acceptance compared to midazolam, however, no significant differences were observed between two groups (59.68% vs 69.23%, RR = 0.85, with 95%CI [0.64, 1.12], *P* = 0.24, *I*^2^ = 76%; Fig 4). The sensitivity analysis indicated that the substantial heterogeneity (*I*^2^ = 76%) was attributable to the Akin *et al*. study [15]. Heterogeneity was resolved (*I*^2^ = 0%) by omitting this study, and the more reliable results indicated that the summary estimate was changed (58.17% vs 78.71%, RR = 0.73, 95% CI [0.64, 0.84], *P* <0.00001). The GRADE summary of findings table indicated that quality of evidence for present outcome was moderate. By the same token, inconsistency (*I*^2^ > 50%) may be considered as main factor (S1 File).

#### Patient’s level of sedation

Five studies with 303 patients described the overall sedation level of pediatric patients. The results of Mitra S *et al*. [14] study indicated that the proportion of patients with satisfactory sedation was slightly higher in the clonidine group compared to midazolam group (100% *vs* 83.33%). Surendar MN *et al*. [18], Saad BB *et al*. [30] and Abusinna RG *et al*. [33] demonstrated that both midazolam and dexmedetomidine provided positive sedative effects, and the proportion of patients with satisfactory sedation was also slightly higher in dexmedetomidine group compared to midazolam group (90.48% *vs* 71.43%, 100.00% *vs* 91.67%, and 78.00% *vs* 70.00%). By contrast, the result of Neville DN *et al*. study indicated that only two patients (11%) were classified as satisfactory sedation in the midazolam group compared to 14 patients (70%) in the dexmedetomidine group.

#### Secondary outcomes

Secondary outcomes including hemodynamic parameters, onset of sedation, recovery time, incidence of postoperative pain needed analgesics rescue and various adverse effects were summarized in Table 2. The details about general hemodynamic parameters including heart rate (HR), systolic blood pressure (SBP), mean arterial pressure (MAP), diastolic blood pressure (DBP), and oxygen saturation were reported separately in 7 studies [17, 20, 24, 26, 28, 32, 34], 4 studies [17, 20, 28, 32], 3 studies [24, 26, 34], 2 studies [28, 32], and 5 studies [17, 20, 24, 26, 34]. The results of general hemodynamic parameters indicated that intranasal dexmedetomidine was associated with significant less value of SBP (MD = 6.97, with 95% CI [0.84, 13.11], *P* = 0.03; *I*^2^ = 94%) and DBP (MD = 6.03, with 95% CI [4.18, 7.87], *P* < 0.0001; *I*^2^ = 19%) compared to midazolam, however, the differences of HR (MD = 4.57, with 95% CI [-2.14, 11.28]) and MAP (MD = 2.32, with 95% CI [-0.96, 5.60]) between two groups were not significant. The differences of “onset of sedation” and “recovery time” between two groups were not significant. The results from analysis of various adverse events indicated that the patients in midazolam group had a higher incidence of nasal irritation/discomfort (32.39% vs 0.00%, RR = 24.00, with 95% CI [3.33, 172.78], *P* < 0.002, *I*^2^ = 0%) and postoperative pain needed analgesics rescue (33.56% vs 16.78%, RR = 2.00, with 95% CI [1.33,3.02], *P* = 0.0009, *I*^2^ = 0%) compared to dexmedetomidine. For incidence of postoperative nauseas or vomiting (PONV) and agitation, the differences between two groups were not different. According to Akin *et al*. study [16], laryngospasm occurred in five children in midazolam group, while none of the dexmedetomidine group patients had such side effect. In addition, as stated by Sheta *et al*. study [19], the incidence of shivering was significantly lower in patients received dexmedetomidine compared with patients received midazolam. The results from GRADE summary of table showed that quality of evidence for most of secondary outcomes was low. Imprecision (lack of events number) and inconsistency (*I*^2^ > 50%) was served as the main reasons. The information was provided in S1 Table.

**Table 2:** Secondary outcomes.

Secondary outcomes	Number of studies (Reference no.)	Patients in Midazolam group (Incidence, %)	Patients in Dexmedetomidine group (Incidence, %)	*I*^2^ (%)	Risk ratio with [95% CI]	*P* value
Postoperative pain needed analgesics rescue	4 (16,19,21, 22)	50/149 (33.56%)	25/149 (16.78%)	0	2.00 [1.33,3.02]	0.0009*
Nauseas and vomiting	3 (16,19,22)	24/114 (21.05%)	20/114 (17.54%)	0	1.20 [0.70, 2.04]	0.5
Agitation	3 (16,19,22)	23/114 (20.18%)	16/114 (14.04%)	51	1.43 [0.59, 3.46]	0.42
Nasal irritation (discomfort)	2 (19,31)	23/71 (32.39%)	0/71 (0.00%)	0	24.00 [3.33, 172.78]	0.002*
Secondary outcomes	Number of studies (Reference no.)	Number of patients in Midazolam group	Number of patients in Dexmedetomidine group	*I*^2^ (%)	Mean difference with [95% CI]	*P* value
Onset of sedation	6 (15,18,19,27,31,33)	262	275	99	-1.05 [-5.15, 3.05]	0.62
Recovery time	4 (18,19,27,31)	172	185	99	-4.08 [-14.35, 6.19]	0.44
Heart rate (HR)	7 (17,20,24,26,28,32,34)	218	218	95	4.57 [-2.14, 11.28]	0.18
Systolic blood pressure (SBP)	4 (17,20,28,32)	127	127	94	6.97 [0.84, 13.11]	0.03*
Mean arterial pressure (MAP)	3 (24,26,34)	91	91	67	2.32 [-0.96, 5.60]	0.17
Diastolic blood pressure (DBP)	2 (28,32)	65	65	19	6.03 [4.18, 7.87]	<0.0001*
Oxygen desaturation	5 (17,20,24,26,34)	153	153	79	-0.19 [-0.60, 0.22]	0.37

## Discussion

The approaches to assess sedation level for pediatric patients vary from different literatures. Level of satisfaction in parental separation before diagnostic and surgical procedure, acceptability of facemask and response to anesthesia induction are often considered as indicators. In addition, various scales are available to provide quantitative data on level of sedation. With good reliability and inter-observer agreement, the Ramsay Sedation Scale (6-points scale) comprising six levels of sedation is identified as an adjunct to practitioner assessment and has been widely used in clinical care [35]. In our present study, such 6-points scale was employed to measuring sedation level of pediatric patients in most of included trials. Additionally, some other scales, such as Richmond Agitation Sedation Scale (a 10-points scale) and University of Michigan Sedation Scale (a 5-points scale), are correlated with Ramsay Sedation Scale and can also be suitable for pediatric population [36]. Given that evaluation scores from sedation scales varied, the differently presented data were not considered in our analysis.

Some studies mentioned the overall sedation level of pediatric patients. However, such outcome data were not combined in analysis for the unclear and inconsistent criteria in presurgical and in pre-procedural sedation. And the results indicated that proportion of patients with satisfactory sedation was higher in both dexmedetomidine and clonidine group compared to midazolam group.

For outcome “Number of patients with satisfactory induction or mask acceptance”, we employed sensitivity analysis by removing particular study to solve the existed heterogeneity and to make the conclusions more reliable. And the co-primary outcomes in present study indicated that the children in group dexmedetomidine were significantly more sedated when they were separated from their parents and when they were experienced induction or mask application compared to group midazolam.

A traditional view is that using of α2-adrenoceptor agonists was always linked with more intense changes of heart rate when compared with other sedatives. But they were still accepted as one appropriate sedative option for pediatric patients in some research [37, 38], because of the great hemodynamic changes could be resolved by decelerating the rate of administration. In our analysis, the results indicated that intranasal dexmedetomidine was associated with significant less value of SBP and DBP compared to midazolam, however, the differences of HR, MAP and oxygen saturation between two groups were not significant. The insufficient studies with small sample size in most secondary outcomes should be served as one limitation in present study, which also might be the reason for inconsistent results of hemodynamics parameters in our analysis.

Additionally, the results of secondary outcomes in our analysis indicated that no difference was found in incidences of some adverse events (agitation and PONV), onset of sedation, and recovery time between two groups. And the outcome also indicated that children received dexmedetomidine as premedication were associated with less incidence of postoperative pain needed analgesics rescue, which illustrated the potential analgesic effects from α2-adrenoceptor agonists and strengthened the previous conclusions [39].

Widespread low or moderate quality in outcomes evaluated by GRADE system may be considered as another limitation in present study. It might be mainly resulted from inconsistency (high heterogeneity) and imprecision (lack of events number). Moreover, researches not published in English or Chinese were excluded in present study as they did not provide sufficient accessible information to our analysis. In our selection process, 12 studies were excluded based on language: 4 were written in Spanish, 3 in German, 2 in French, 1 in Italian, 1 in Turkish, and 1 in Korean, then language bias cannot be excluded. To compensate for absence of information resource, a thorough search strategy and an additional source from Google scholar were considered by us, however, the number of enrolled pediatric patients was still deficient. Especially, the studies on comparison between intranasal clonidine and intranasal midazolam are inadequate. After literature screening and full text perusing, only one study compared midazolam with clonidine met the inclusion criteria of present study, and majority of researches still focuses on other drug delivery routes [40, 41]. Therefore, outcome data from study focused on intranasal clonidine versus intranasal midazolam were not combined in analysis. And the current evidences also indicated that proportion of patients with satisfactory sedation was slightly higher in the clonidine group compared to midazolam group. Moreover, owing to all outcomes involved fewer than ten studies, the Begg’s and Egger’s test to evaluate the publication bias were not taken into account in present study [42].

## Conclusion

Compared with midazolam, dexmedetomidine should be considered as the preferred intranasal sedative option for pediatric patients, since it provides more satisfactory sedative level with less incidence of postoperative pain and nasal irritation. However, the evidences about intranasal clonidine versus intranasal midazolam were still insufficient. And overall low and moderate quality evidences evaluated by GRADE system suggest that superiority of intranasal α2-adrenoceptor agonists in pediatric sedation needs to be validated by more future studies with high quality and large sample size.

## Supporting information

S1 File Search strategy. (DOCX)

S1 Table GRADE summary of findings table. (DOCX)

S1 Checklist PRISMA checklist. (DOCX)
