# Supplementary material for: A comparative evaluation of intranasal α2-adrenoceptor agonists and intranasal midazolam as premedication in pediatric sedation: A meta-analysis of randomized controlled trials
Source: PLoS One. 2023 Feb 14;18(2):e0281751. doi: 10.1371/journal.pone.0281751 (PMC9928077; doi:10.1371/journal.pone.0281751)
Supplement: S1 Table — (DOCX) [file pone.0281751.s002.docx]

**Table S1. GRADE summary of findings table**

| **Quality assessment** | | | | | | | **Summary of Findings** | | | | |
| --- | --- | --- | --- | --- | --- | --- | --- | --- | --- | --- | --- |
| **Participants (studies) Follow up** | **Risk of bias** | **Inconsistency** | **Indirectness** | **Imprecision** | **Publication bias** | **Overall quality of evidence** | **Study event rates (%)** | | **Relative effect** (95% CI) | **Anticipated absolute effects** | |
|  |  |  |  |  |  |  | **With Control** | **With Satisfactory separation from parents** |  | **Risk with Control** | **Risk difference with Satisfactory separation from parents** (95% CI) |
| **Primary outcomes** | | | | | | | | | | | |
| **Satisfactory separation from parents** (CRITICAL OUTCOME) | | | | | | | | | | | |
| 556 (8 studies) | no serious risk of bias | serious1 | no serious indirectness | no serious imprecision | undetected | **ÅÅÅO** **MODERATE** | 209/278  (75.2%) | 147/278  (52.9%) | **RR 0.7**  (0.55 to 0.9) | **Study population** | |
|  |  |  |  |  |  |  |  |  |  | **752 per 1000** | **226 fewer per 1000** (from 75 fewer to 338 fewer) |
|  |  |  |  |  |  |  |  |  |  | **Moderate** | |
|  |  |  |  |  |  |  |  |  |  | **789 per 1000** | **237 fewer per 1000** (from 79 fewer to 355 fewer) |

1 *I*2 > 50%

| **Quality assessment** | | | | | | | **Summary of Findings** | | | | |
| --- | --- | --- | --- | --- | --- | --- | --- | --- | --- | --- | --- |
| **Satisfactory induction or mask acceptance** (CRITICAL OUTCOME) | | | | | | | | | | | |
| 500 (7 studies) | no serious risk of bias | serious1 | no serious indirectness | no serious imprecision | undetected | **ÅÅÅO MODERATE** | 171/247  (69.2%) | 151/253  (59.7%) | **RR 0.85**  (0.64 to 1.12) | **Study population** | |
|  |  |  |  |  |  |  |  |  |  | **692 per 1000** | **104 fewer per 1000** (from 249 fewer to 83 more) |
|  |  |  |  |  |  |  |  |  |  | **Moderate** | |
|  |  |  |  |  |  |  |  |  |  | **767 per 1000** | **115 fewer per 1000** (from 276 fewer to 92 more) |

1 *I*2 > 50%

| **Quality assessment** | | | | | | | **Summary of Findings** | | | | |
| --- | --- | --- | --- | --- | --- | --- | --- | --- | --- | --- | --- |
| **Participants (studies) Follow up** | **Risk of bias** | **Inconsistency** | **Indirectness** | **Imprecision** | **Publication bias** | **Overall quality of evidence** | **Study event rates (%)** | | **Relative effect** (95% CI) | **Anticipated absolute effects** | |
|  |  |  |  |  |  |  | **With Control** | **With Incidence of postoperative pain needed analgesics rescue** |  | **Risk with Control** | **Risk difference with Incidence of postoperative pain needed analgesics rescue** (95% CI) |
| **Incidence of postoperative pain needed analgesics rescue** (IMPORTANT OUTCOME) | | | | | | | | | | | |
| 298 (4 studies) | no serious risk of bias | no serious inconsistency | no serious indirectness | serious1 | undetected | **ÅÅÅO** **MODERATE** | 25/149  (16.8%) | 50/149  (33.6%) | **RR 2**  (1.33 to 3.02) | **Study population** | |
|  |  |  |  |  |  |  |  |  |  | **168 per 1000** | **168 more per 1000** (from 55 more to 339 more) |
|  |  |  |  |  |  |  |  |  |  | **Moderate** | |
|  |  |  |  |  |  |  |  |  |  | **124 per 1000** | **124 more per 1000** (from 41 more to 250 more) |

1 Total number of events is less than 300

| **Quality assessment** | | | | | | | **Summary of Findings** | | | | |
| --- | --- | --- | --- | --- | --- | --- | --- | --- | --- | --- | --- |
| **Participants (studies) Follow up** | **Risk of bias** | **Inconsistency** | **Indirectness** | **Imprecision** | **Publication bias** | **Overall quality of evidence** | **Study event rates (%)** | | **Relative effect** (95% CI) | **Anticipated absolute effects** | |
|  |  |  |  |  |  |  | **With Control** | **With Onset of sedation** |  | **Risk with Control** | **Risk difference with Onset of sedation** (95% CI) |
| **Onset of sedation** (IMPORTANT OUTCOME; Better indicated by lower values) | | | | | | | | | | | |
| 537 (6 studies) | no serious risk of bias | very serious1 | no serious indirectness | no serious imprecision | undetected | **ÅÅOO** **LOW** | 275 | 262 | **-** |  | The mean onset of sedation in the intervention groups was **1.05 lower** (5.15 lower to 3.05 higher) |

1 *I*2 > 50%

| **Quality assessment** | | | | | | | **Summary of Findings** | | | | |
| --- | --- | --- | --- | --- | --- | --- | --- | --- | --- | --- | --- |
| **Participants (studies) Follow up** | **Risk of bias** | **Inconsistency** | **Indirectness** | **Imprecision** | **Publication bias** | **Overall quality of evidence** | **Study event rates (%)** | | **Relative effect** (95% CI) | **Anticipated absolute effects** | |
|  |  |  |  |  |  |  | **With Control** | **With Recovery time** |  | **Risk with Control** | **Risk difference with Recovery time** (95% CI) |
| **Recovery time** (IMPORTANT OUTCOME; Better indicated by lower values) | | | | | | | | | | | |
| 357 (4 studies) | no serious risk of bias | very serious1 | no serious indirectness | serious2 | undetected | **ÅOOO** **VERY LOW** | 185 | 172 | **-** |  | The mean recovery time in the intervention groups was **4.08 lower** (14.35 lower to 6.19 higher) |

1 *I*2 > 50%
2 The total number of patients is less than 400

| **Quality assessment** | | | | | | | **Summary of Findings** | | | | |
| --- | --- | --- | --- | --- | --- | --- | --- | --- | --- | --- | --- |
| **Participants (studies) Follow up** | **Risk of bias** | **Inconsistency** | **Indirectness** | **Imprecision** | **Publication bias** | **Overall quality of evidence** | **Study event rates (%)** | | **Relative effect** (95% CI) | **Anticipated absolute effects** | |
|  |  |  |  |  |  |  | **With Control** | **With Complications and side effects: Nauseas and vomiting** |  | **Risk with Control** | **Risk difference with Complications and side effects: Nauseas and vomiting** (95% CI) |
| **Complications and side effects: Nauseas and vomiting** (IMPORTANT OUTCOME) | | | | | | | | | | | |
| 228 (3 studies) | no serious risk of bias | no serious inconsistency | no serious indirectness | very serious1 | undetected | **ÅÅOO** **LOW** | 20/114  (17.5%) | 24/114  (21.1%) | **RR 1.2**  (0.7 to 2.04) | **Study population** | |
|  |  |  |  |  |  |  |  |  |  | **175 per 1000** | **35 more per 1000** (from 53 fewer to 182 more) |
|  |  |  |  |  |  |  |  |  |  | **Moderate** | |
|  |  |  |  |  |  |  |  |  |  | **167 per 1000** | **33 more per 1000** (from 50 fewer to 174 more) |
| **Complications and side effects: Agitation** (IMPORTANT OUTCOME) | | | | | | | | | | | |
| 228 (3 studies) | no serious risk of bias | serious2 | no serious indirectness | very serious3 | undetected | **ÅOOO** **VERY LOW** | 16/114  (14%) | 23/114  (20.2%) | **RR 1.43**  (0.59 to 3.46) | **Study population** | |
|  |  |  |  |  |  |  |  |  |  | **140 per 1000** | **60 more per 1000** (from 58 fewer to 345 more) |
|  |  |  |  |  |  |  |  |  |  | **Moderate** | |
|  |  |  |  |  |  |  |  |  |  | **121 per 1000** | **52 more per 1000** (from 50 fewer to 298 more) |
| **Complications and side effects: Nasal irritation (discomfort)** (IMPORTANT OUTCOME) | | | | | | | | | | | |
| 142 (2 studies) | no serious risk of bias | no serious inconsistency | no serious indirectness | very serious3 | undetected | **ÅÅOO** **LOW** | 0/71  (0%) | 23/71  (32.4%) | **RR 24**  (3.33 to 172.78) | **Study population** | |
|  |  |  |  |  |  |  |  |  |  | **0 per 1000** | **-** |
|  |  |  |  |  |  |  |  |  |  | **Moderate** | |
|  |  |  |  |  |  |  |  |  |  | **0 per 1000** | **-** |

1 Total number of events is less than 300
2 *I*2 > 50%
3 Total number of events is less than 100

| **Quality assessment** | | | | | | | **Summary of Findings** | | | | |
| --- | --- | --- | --- | --- | --- | --- | --- | --- | --- | --- | --- |
| **Participants (studies) Follow up** | **Risk of bias** | **Inconsistency** | **Indirectness** | **Imprecision** | **Publication bias** | **Overall quality of evidence** | **Study event rates (%)** | | **Relative effect** (95% CI) | **Anticipated absolute effects** | |
|  |  |  |  |  |  |  | **With Control** | **With Hemodynamic status** |  | **Risk with Control** | **Risk difference with Hemodynamic status** (95% CI) |
| **Hemodynamic status (HR)** (IMPORTANT OUTCOME; Better indicated by lower values) | | | | | | | | | | | |
| 436 (7 studies) | no serious risk of bias | very serious1 | no serious indirectness | no serious imprecision | undetected | **ÅÅOO** **LOW** | 218 | 218 | **-** |  | The mean hemodynamic status (hr) in the intervention groups was **4.57 higher** (2.14 lower to 11.28 higher) |
| **Hemodynamic status (SBP)** (IMPORTANT OUTCOME; Better indicated by lower values) | | | | | | | | | | | |
| 254 (4 studies) | no serious risk of bias | very serious1 | no serious indirectness | serious2 | undetected | **ÅOOO** **VERY LOW** | 127 | 127 | **-** |  | The mean hemodynamic status (sbp) in the intervention groups was **6.97 higher** (0.84 to 13.11 higher) |
| **Hemodynamic status (MBP)** (IMPORTANT OUTCOME; Better indicated by lower values) | | | | | | | | | | | |
| 182 (3 studies) | no serious risk of bias | serious1 | no serious indirectness | serious2 | undetected | **ÅÅOO** **LOW** | 91 | 91 | **-** |  | The mean hemodynamic status (mbp) in the intervention groups was **2.32 higher** (0.96 lower to 5.6 higher) |
| **Hemodynamic status (DBP)** (IMPORTANT OUTCOME; Better indicated by lower values) | | | | | | | | | | | |
| 130 (2 studies) | no serious risk of bias | no serious inconsistency | no serious indirectness | very serious2 | undetected | **ÅÅOO** **LOW** | 65 | 65 | **-** |  | The mean hemodynamic status (dbp) in the intervention groups was **6.03 higher** (4.18 to 7.87 higher) |
| **Oxygen saturation** (IMPORTANT OUTCOME; Better indicated by lower values) | | | | | | | | | | | |
| 306 (5 studies) | no serious risk of bias | serious1 | no serious indirectness | serious2 | undetected | **ÅÅOO** **LOW** | 153 | 153 | **-** |  | The mean oxygen saturation in the intervention groups was **0.19 lower** (0.6 lower to 0.22 higher) |

1 *I*2 > 50%
2 The total number of patients is less than 400
